# Cross-sectional survey of knowledge of obstetric danger signs among women in rural Madagascar

**DOI:** 10.1186/s12884-018-1664-x

**Published:** 2018-02-05

**Authors:** Ania Salem, Oriane Lacour, Stefano Scaringella, Josea Herinianasolo, Anne Caroline Benski, Giovanna Stancanelli, Pierre Vassilakos, Patrick Petignat, Nicole Christine Schmidt

**Affiliations:** 1 Faculty of Medicine, University of Geneva, Geneva, Switzerland; 2 Department of Obstetrics and Gynecology, University Hospitals of Geneva, Geneva, Switzerland; 3 Centre Médico-chirurgical Saint Damien, Ambanja, Madagascar; 4 AISPO, Associazione Italiana Solidarietà tra I Popoli, Milan, Italy

**Keywords:** Obstetric danger signs, Pregnancy, Childbirth, Postpartum, Newborn, mHealth, Madagascar

## Abstract

**Background:**

Antenatal care (ANC) has the potential to identify and manage obstetric complications, educate women about risks during pregnancy and promote skilled birth attendance during childbirth. The aim of this study was to assess women’s knowledge of obstetric danger signs and factors associated with this knowledge in Ambanja, Madagascar. It also sought to evaluate whether the participation in a mobile health (mHealth) project that aimed to provide comprehensive ANC to pregnant women in remote areas influenced women’s knowledge of obstetric danger signs.

**Methods:**

From April to October 2015, a non-random, convenience sample of 372 women in their first year postpartum were recruited, including 161 who had participated in the mHealth project. Data were analyzed using bivariate and multivariate logistic regression.

**Results:**

Knowledge of at least one danger sign varied from 80.9% of women knowing danger sign(s) in pregnancy, to 51.9%, 50.8% and 53.2% at delivery, postpartum and in the newborn, respectively. Participation in the mHealth intervention, higher household income, and receipt of information about danger signs during pregnancy were associated with knowledge of danger signs during delivery, in bivariate analysis; only higher household income and mHealth project participation were independently associated. Higher educational attainment and receipt of information about danger signs in antenatal care were associated with significantly higher odds of knowing danger sign(s) for the newborn in both bivariate and multivariate analysis.

**Conclusions:**

Knowledge of obstetric danger signs is low. Information provision during pregnancy and with mHealth is promising.

**Trial registration:**

This trial was retrospectively registered at the International Standard Randomized Controlled Trial Register (identifier ISRCTN15798183; August 22, 2015).

**Electronic supplementary material:**

The online version of this article (10.1186/s12884-018-1664-x) contains supplementary material, which is available to authorized users.

## Background

Maternal mortality remains high in low- and middle-income country populations. In 2015 the global maternal mortality rate declined to 216 maternal deaths/100,000 live births. Most women worldwide died due to complications of pregnancy or childbirth; most of them in Sub-Saharan Africa, where the maternal mortality rate remained high with 546 deaths/100,000 live births [1]. In Madagascar, the estimated maternal mortality rate in 2015 was 353 deaths/100,000 live births [2].

Approximately 80% of maternal deaths worldwide are due to direct complications during pregnancy such as severe haemorrhage, obstructed labor, infections, pregnancy-related hypertension, and/or unsafe induced abortion. Indirect causes such as diabetes, malaria or anaemia can worsen the mother’s condition during pregnancy and also lead to maternal death. Most of these maternal deaths could be avoided by providing comprehensive antenatal care, skilled delivery care and access to emergency obstetric care [3–5].

Previous studies have shown a positive association between the knowledge of danger signs before, during or after delivery, and institutional delivery [6, 7]. Karkee and colleagues reported that women who could spontaneously mention any danger sign during the antepartum, intrapartum or postpartum period were more likely to deliver in a health facility [6]. Similarly, in the study conducted by Hailu and colleagues in Aleta Wondo district, Ethiopian women who received maternal and child health education were nine times more likely to deliver in a health facility [7]. Moreover, as shown by Bogale and colleagues, women who attend antenatal care (ANC) are more likely to know obstetric danger signs during pregnancy and delivery [8]. This is in line with the World Health Organization (WHO) recommendations to raise awareness among women about danger signs before, during or after delivery to improve early detection of problems and reduce the delay to seek obstetric care [9]. Therefore, ANC provides a unique opportunity to strengthen knowledge of obstetric danger signs and encourage institutional delivery.

In Madagascar, according to the Demographic Health Survey 2012–2013, 82.1% of mothers had at least one ANC visit, but only 44% delivered with a skilled birth attendant and only 37.9% in a healthcare facility [10]. Reaching women in remote areas is one of the aims of the Pregnancy-And-Newborn Diagnostic-Assessment (PANDA) project that begun in January 2015 at the Centre Médico-Chirurgical (CMC) Saint Damien, Ambanja, Madagascar. PANDA is a telemedicine system based on mobile technology that incorporates the WHO recommendations for antenatal care (ANC). The system includes:

- A smartphone with an Android icon-based application to collect personal and clinical patient data. Furthermore, it includes a health education guide with a focus on birth preparedness including danger signs during pregnancy childbirth and in the newborn.
- The so called “PANDA point of care” that contains a solar backpack with photovoltaic power including the diagnostic devices to test for example for blood pressure, fever etc.
- The “PANDA medical unit” which is a JAVA-based software system that is hosted in the referral hospital and allows doctors to check the data and create a clinical chart with individual patient data.

Further details on the usability and feasibility of the PANDA have been published recently [5].

The objectives of the study were to assess the prevalence of knowledge of obstetric danger signs and identify predictors of knowledge of obstetric danger signs.

## Methods

The cross-sectional study was conducted from August to October 2015 in the district of Ambanja, approximately 500 km from Antananarivo, the capital of Madagascar. This rural area has an estimated population of 200,000 and is economically dependent on farming. The study was based at the CMC Saint Damien, a private non-profit clinic founded in 1988, which collaborates with the Health Ministry. The CMC provides ANC for the urban population and at 18 dispensaries in Ambanja district in a radius of 250 km.

In this study, a non-random, convenience sample of 372 women was recruited at the CMC Saint Damian and in the surrounding villages during health visits and health campaigns. Among the 372 women, 161 had participated previously in ANC provided by the PANDA mHealth system and 211 had not. Women were eligible to participate regardless of their age if they were in their first year postpartum. The reason to exclude women who delivered more than 12 months ago was to reduce recall bias. Eligible women participated after having given written consent by signature or fingerprint in an interviewer-administered 74-item, translated after pretesting from English into French and Malagasy (see Additional file 1). Additionally, Sakalava-speaking interpreters assisted the co-investigators in the recruitment of participants. Investigators and translators signed a written document committing to respect participants’ confidentiality and anonymity prior to the study. The questionnaire assessed the following five categories:

- Maternal factors: e.g. sociodemographic factors and obstetric history.
- Social factors: e.g. characteristics of partner and family but also of the community.
- Macro factors: e.g. distance to and hours of operation of the healthcare facility, availability of transport, and fees.
- Facility factors: e.g. previous experiences with the health system.
- Obstetric knowledge: participants were asked to name spontaneously the most common danger signs during pregnancy, delivery, and the postpartum periods for the mother and the newborn. Their answers were then matched to a list of key obstetric danger signs defined for each phase (Table 1).

**Table 1:** Key Obstetric danger signs

Pregnancy	
Vaginal Bleeding	
Swollen hands and body	
Loss of consciousness and convulsions	
Blurry vision	
Violent headache or vertigo	
Fever	
Acute abdominal pain	
Absence of fetal movement	
Labor and Delivery	
Prolonged labor (> 12 h)	
Retained placenta > 30 min after delivery	
Loss of consciousness and convulsions	
Postpartum (mother)	
Fever	
Foul-smelling amniotic fluid or vaginal discharge	
Swollen hands and body	
Loss of consciousness and convulsions	
Post-partum (newborn)	
Doesn’t suckle, difficulty eating or vomiting	
Difficulty breathing	
Is blue	
Is cold or hot or has high fever	
Has skin eruption	
Is very small	

After completion of the questionnaire, each woman was provided a short individual educational session of approximately five minutes duration about danger signs for mother and child in the ante-, intra- and postpartum period using a small card developed in collaboration with “Enfants du Monde”. One side of the card showed images to suggest maternal danger signs such as maternal bleeding, blurry vision, fever and convulsions. The other side displayed risk signs of the newborn like fever or hypothermia, convulsion, omphalitis, inability to breastfeed, and breathing difficulties.

Data were collected and checked by the principal investigator for completeness and corrective measures were taken if necessary. Data were coded, entered, cleaned and analyzed using Stata Data Analysis and Statistical Software Version 13 (Stata Corporation, College Station, TX, USA). The dependent variable knowledge of obstetric danger signs was defined when a woman mentioned unprompted at least one or more of the key danger signs that are outlined in Table 1. The baseline factors such as age, marital status, education or parity, which are associated with the knowledge of danger signs, were compared either as discrete variables using contingency table chi-squared tests or as continuous variables using two-sample t-tests. Statistical significance was considered as *p*-value <.05. Binary and multivariable logistic regression analyses were carried out to identify factors associated with the knowledge of danger signs. Variables that were significant in the bivariate analysis were entered into the multiple logistic regression analysis. To estimate the association of key danger signs during the four periods (pregnancy, delivery, postpartum and in the newborn) and each independent variable odds ratio (OR) with 95% confidence intervals (CI). A CI that did not overlap 1 was considered statistically significant.

The research adhered to the STROBE guidelines for cross-sectional studies.

## Results

A total of 372 women aged between 13 and 45 years consented to participation and were interviewed. Most were single (68.0%), living in rural areas (68.8%), and secondary school entrants (53.8%); 43.0% had not started the last three years of secondary school (Table 2). The main professions reported were farmer (33.3%) or housewife (47.0%). Economic status was relatively low with most having a monthly household income of less than 100,000 Ariary (approximately 28 Euros); 77.4% did not have electricity at home and 51.3% had no cell phone. For 85.7%, a dispensary or hospital was less than an hour’s walk away.

**Table 2:** Socio-demographic and reproductive health characteristics (*N* = 372)

	Total (n)	Total (%)
Place of residence		
Rural	256	68.8
Urban	116	31.2
Age		
< 19	100	26.9
20–24	87	23.4
25–29	72	19.4
> 30	112	30.2
Status		
Married or Living with a partner	101	27.1
Single or House-sharing	253	68.0
Separated or Divorced	18	4.8
Education		
None	56	15.1
Primary	102	27.4
Secondary	200	53.8
Tertiary	14	3.8
Profession		
Housewife	175	47.0
Farmer	124	33.3
Merchant	43	11.6
Other	30	8.1
Income		
< 100′000 Ar	191	51.3
100′000–300′000 Ar	142	38.2
> 300′000 Ar	39	10.48
Electricity		
Yes	84	22.6
No	288	77.4
Cell phone		
Yes	181	48.7
No	191	51.3
Walking distance to Hospital or Dispensary		
< 1 h	318	85.7
> 1 h	53	14.3
Pregnancies		
1	113	30.4
2 to 4	184	49.6
> 5	75	20.2
Parity		
1	128	34.5
2 to 4	184	49.6
> 5	59	15.9
Ever had a stillbirth		
Yes	16	4.3
No	356	95.7
Number of Miscarriages		
0	331	89.0
1	32	8.6
> 1	9	2.4
Number of ANC visits during last pregnancy		
0	3	0.8
1	8	2.2
2 to 3	104	27.9
≥ 4	257	69.1
Advised site for delivery		
Hospital	180	55.4
Dispensary	142	43.7
Other	3	0.9
Pregnancy Intendedness		
Desired now	257	69.1
Mistimed	74	19.9
Unwanted	41	11.0
Participation in PANDA mHealth intervention		
Yes	161	43.3
No	211	56.7
Received information about problems during pregnancy (*N* = 370)		
Yes	262	70.8
No	108	29.2

Most of the participants were multiparous (65.6%) and reported two to four previous deliveries (Table 2). Thirty-one percent of women stated that their pregnancy was unintended, either mistimed (19.9%) or not wanted at all (11.0%).

Almost all women had had at least one ANC visit (99.2%). Most (69.6%) had attended at least the WHO-recommended four ANC visits. Many women stated that they received information during ANC about potential problems that might occur during pregnancy and delivery (70.8%), and nearly all were advised to deliver at the hospital (55.4%) or dispensary (43.7%). Healthcare workers were the main source of this information (60.8%). Few women received information from friends, neighbors or family members (6.2%).

**Fig. 1:**
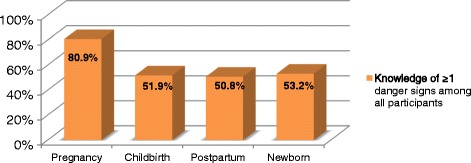
Percentage of women who could spontaneously mention ≥1 key danger sign(s)

Most participants agreed that a pregnancy could be dangerous for a woman’s health (*n* = 326, 87.6%). Almost all participants (77.2%) reported that health facilities were available in their vicinity and only 14.5% stated that the nearest health facility was more than a one-hour walk from their home. Most women could mention at least one or more key danger signs during pregnancy (80.9%). The most frequently mentioned key danger signs for pregnancy were fever (41.1%), headache (32.0%), swollen hands and body (28.8%), and vaginal bleeding (26.9%). For the other three periods (delivery, postpartum and neonatal) knowledge of at least one danger sign was lower (51.9%, 50.8%, and 53.2%, respectively) (Fig. 1).

In the bivariate analysis, higher income, PANDA mHealth project participation and receipt of information about danger signs were associated with significantly higher odds of knowledge of danger signs at delivery (Table 3).

**Table 3:** Factors associated with the knowledge of danger signs during delivery

	CRUDE OR (95% Cl)	Adjusted OR (95% Cl)
Age		
< 19 years	1.00	1.00
20–29 years	1.44 (0.87–2.38)	1.28 (0.73–2.23)
> 30 years	1.52 (0.88–2.61)	1.34 (0.72–2.49)
Marital Status		
Single	1.00	1.00
Married or living with partner	1.51 (0.95–2.39)	1.43 (0.84–2.44)
Separated or divorced	0.60 (0.21–1.72)	0.75 (0.25–2.27)
Education		
No education	1.00	1.00
Primary education only	1.07 (0.56–2.05)	1.08 (0.53–2.18)
Secondary education	1.35 (0.75–2.45)	1.26 (0.65–2.46)
Tertiary education	2.88 (0.81–10.30)	1.60 (0.43–6.09)
Information received about danger signs during pregnancy?		
Yes	2.18 (1.43–3.31)**	1.50 (0.90–2.49)
No	1.00	1.00
Household income		
< 28 Euros	1.00	1.00
≥ 28 Euros	2.31 (1.52–3.50)**	1.90 (1.21–2.97)**
Participation in the Pregnancy-And-Newborn Diagnostic-Assessment (PANDA)		
Yes	2.18 (1.43–3.31)**	1.83 (1.15–2.91)*
No	1.00	1.00

*Significant at *p* < 0.05

**Significant at *p* < 0.01

There was no significant difference in the knowledge of danger signs during pregnancy or in the postpartum period between both groups.

However, higher household income and PANDA participation were the only factors that emerged as significant independent predictors of knowing danger signs during delivery in the multivariate analysis.

Higher educational attainment and maternal receipt of information about danger signs were associated with significantly higher odds of knowledge of danger signs in the neonate in both bivariate and multivariate analysis (Table 4).

**Table 4:** Factors associated with the knowledge of danger signs for the newborn

	CRUDE OR (95% Cl)	Adjusted OR (95% Cl)
Age		
< 19 years	1.00	1.00
20–29 years	0.97 (0.59–1.60)	0.89 (0.51–1.56)
> 30 years	1.43 (0.83–2.47)	1–72)0.91–3.27)
Marital Status		
Single	1.00	1.00
Married or living with partner	1.23 (0–78-196)	0.66 (0.95–2.79)
Separated or divorced	0.49 (0.17–1.41)	0.66 (0.21–2.07)
Education		
No education	1.00	1.00
Primary education only	2.93 (1.46–5.88)**	3.37 (1.59–7.19)**
Secondary education	3.38 (1.78–6.44)**	4.34 (2.10–8.95)**
Tertiary education	15.00 (3.01–74.69)**	16.96 (3.18–90.7)**
Information received about danger signs during pregnancy?		
Yes	2.05 (1.58–3.97)**	2.09 (1.24–3.51)**
No	1.00	1.00
Household income		
< 100′000 Malagasy Arias	1.00	1.00
> 100′000 Malagasy Arias	0.73 (0.46–1.16)	0.73 (0.46–1.16)
PANDA participation		
Yes	1.07 (0.71–1.61)	1.37 (0.85–2.)
No	1.00	1.00

*Significant at *p* < 0.05

**Significant at *p* < 0.01

**Fig. 2:**
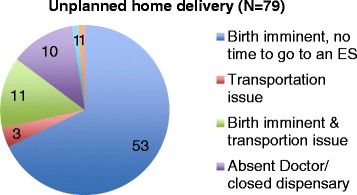
Reasons for unplanned home delivery

Among the 372 women who participated in the study, 144 (38.7%) delivered in a hospital and 131 (35.2%) in a dispensary. Only 92 (24.7%) delivered at home. Importantly only 13 of those women (0.03%) reported a planned home birth. The main reasons for an unplanned home birth were precipitous labor and transportation issues (*n* = 64/79; 81.0%). However, in ten cases the reason was a closed health facility or the absence of doctor or midwife (Fig. 2). As very few women in our study had a planned home birth, it was not possible to analyze determinants influencing home birth.

## Discussion

The knowledge of danger signs during pregnancy, childbirth, and in the postpartum and neonatal periods is essential for seeking medical help if necessary.

In our study the knowledge of danger signs for the four periods showed important variations. This finding is consistent with other studies in Africa [7, 8, 11–13]. In a study conducted in Uganda, out of 764 women 52% mentioned at least one danger sign during pregnancy, 72% during childbirth and 72% during the postpartum [11]. Similarly, in a study conducted in Tanzania, 26% of 1118 women knew at least one danger sign during pregnancy, 23% during delivery and 40% after delivery [12].

Despite the variations in the knowledge about danger signs during the different periods, which are consistent with our studies, we noted important discrepancies in respect to the period of pregnancy or childbirth in which women were most knowledgeable. In our study, more than 80% of women knew at least one danger sign during pregnancy, which is similar to a study conducted in Kenya, where 67% out of 394 women new at least one danger sign during pregnancy [14]. Only half of the women in our study knew at least one danger sign during the other periods.

In contrast to our findings, in the study conducted in Uganda the knowledge of at least one danger sign was best during childbirth and the postpartum, while in the study in Tanzania, women were most knowledgeable for danger signs after delivery [11, 12]. In the studies conducted by Hailu et al. in two different districts in Ethiopia, the knowledge of at least two danger signs was highest during childbirth, while in the study in Goba district Ethiopia, most of the 179 participants were most knowledgeable for danger signs during pregnancy (31.9%) [7, 8, 13].

In the further discussion, we aim to find possible explanations, why:

1. the percentage of women who are knowledgeable of danger signs varies importantly among different studies.
2. the knowledge of danger signs during pregnancy was highest in our study in contrast to other studies conducted in African countries.

First of all, the cited studies used different methodologies, an important factor explaining the different percentages in the knowledge of danger signs. Bogale et al. and Hailu and colleagues for example only noted a positive obstetric awareness if at least two or three obstetric danger signs were identified, while in our and two other studies the knowledge of at least one danger sign was considered as knowledgeable [7, 8, 11–13].

Furthermore, educational status of women varied in the different studies. Higher education status has been associated with increased obstetric awareness. Having secondary education or higher increased the knowledge of danger signs during pregnancy, delivery and the postpartum significantly [12]. However, the educational status of the mothers was not statistically significant in all the studies. Even if in the study conducted in Tsegedie district formal education of the mothers was strongly associated with the knowledge about danger signs during both pregnancy and delivery, in the study conducted in Goba district, Ethiopia, a statistical significant association was found for danger signs only during delivery [8, 13]. This is in concordance with our study in which the educational status was not statistically significant during all periods: it was only positively associated with the knowledge of danger signs in the newborn; during delivery and the postpartum it was not and during childbirth it showed only a synergistic direction. One possible explanation for this discrepancy might be that the level of education differs in each study population. Most participants in our study had a relatively high educational status compared to those in other studies in similar low- and middle-income African countries. In our study, only 15.0% of women had not attended primary school and more than half of the women were secondary school entrants. In contrast, in the study conducted by Hailu and colleagues in Tsegedie district, Ethiopia, 54.4% of women were unable to read [13].

However, the different education status of women does not explain why in our study women were most knowledgeable for danger signs during pregnancy. The most frequently mentioned key danger signs for pregnancy were fever (41.1%), headache (32.0%), swollen hands and body (28.8%), and vaginal bleeding (26.9%). This is also in contrast to other studies, in which the most common mentioned danger sign during pregnancy was vaginal bleeding. In the study in Tsegedie district, Ethiopia, among 485 women, 49.1% mentioned vaginal bleeding as a danger sign during pregnancy followed by 41.6% of women who mentioned swelling of leg or face [13]. Severe vaginal bleeding was also the most common mentioned danger sign during pregnancy in the studies conducted in Uganda and in Goba district, Ethiopia [8, 11].

A possible explanation for this difference might the positive effect of a public health campaign for the prevention of preeclampsia recently conducted in the study area of Ambanja. The campaign highlighted the danger signs for preeclampsia during pregnancy and didn’t mention explicitly danger signs during childbirth, in the postpartum or in the newborn. This could explain the lower knowledge of danger signs among women during those periods.

Since at least 20% of maternal deaths occur due to complications during delivery and the immediate postpartum period (such as hemorrhage or infection), information about postpartum problems is essential to save mother’s lives [1]. Also, the knowledge of danger signs in the newborn was lower in our study than in other studies conducted in Africa [15, 16]. Madagascar’s newborn mortality rate remains high and early detection of neonatal illness to improve newborn survival is urgently needed [17].

In our study we observed a synergistic effect of education. We agree with authors who have previously stated that educated women might have better access to information and that education facilitates the understanding of health information and improves autonomy in health decision-making [12, 13].

However, information about danger signs should be provided additionally, because in our opinion, the recent conducted health campaign influenced positively the knowledge about danger signs of preeclampsia during pregnancy. Furthermore, the receipt of health information was independently associated with higher odds of knowledge of at least of danger sign for the newborn and of danger signs during delivery. Even if Cochrane review published in 2007 failed to establish the effectiveness of ANC education for childbirth, the results of the cited studies and our findings demonstrate the challenge of providing high quality information during ANC [18]. As previously noted by Pembe and colleagues, individual counseling times are often short and influenced by the complex interaction between patient and health care provider, as well as socio-cultural aspects [12].

As nearly all participants reported at least one ANC visit, ANC provides a unique opportunity to educate women about obstetric danger signs. The challenges to providing quality ANC should be considered in current and future programs, including the important role that an mHealth application may have on these services. mHealth interventions in low- and middle income countries have increased in recent years and most of the interventions used by health care providers in the field of maternal and neonatal health have addressed health education, workflow and disease prevention [19]. The multivariate analysis showed the promising tendency that participation in the PANDA mHealth application program was independently associated with women’s knowledge of danger signs at delivery. However, participation in the mHealth program did not improve women’s knowledge of danger signs during the other periods. One possible explanation for this observation is that a recently conducted public health campaign about danger signs during pregnancy increased women’s knowledge in both groups. Furthermore, the interaction time of the educational section of the PANDA mHealth intervention was short, which might explain no improvement of knowledge for example in the newborn. Lastly, knowledge around danger signs for the postpartum period was not strengthened sufficiently.

Another important point of consideration is, given the high attendance at the ANC visits, whether family planning counseling should be included in the ANC visits in Ambanja, Madagascar. As previously noted, nearly all women in our study had benefited from ANC during their pregnancy and more than two thirds (69.7%) had even attended the WHO-recommended four regular ANC visits. Furthermore, we believe that the women reached in our study were in general those who had a more positive attitude towards the health system. However, the rate of unintended pregnancies in our sample was higher than reported in the Demographic Health Survey in 2009 and more similar to the rates reported by the Guttmacher Foundation for the African Continent [21, 22]. Timing pregnancies has been linked to a reduction in maternal and child mortality; therefore, the inclusion of family planning into ANC has been recommended [20, 21]. Even if in a recent review the mixed results exploring the relationship of ANC visits on contraceptive uptake in the first year postpartum have been highlighted, it has been mentioned as well that some studies conducted in Kenya and South Africa have shown an increased uptake of contraceptive methods in the first year postpartum when counseling during ANC visits was provided [22, 23]. As the provision of family planning reduces maternal deaths due to unsafe abortions and spacing of pregnancies can improve the survival chances and health outcomes of women and newborn, we consider the inclusion of family planning into ANC as important to consider in our setting [24].

Predictors of knowledge of danger signs are important, as previous studies have linked this knowledge to institutional delivery [6, 7]. A study conducted in Nepal by Karkee and colleagues found that the acknowledgment of potential unexpected problems during pregnancy and childbirth was associated with almost 6-fold higher odds of institutional delivery [6]. In our study it was not possible to test this correlation, as only 13 women had a planned home birth. This is in sharp contrast to the data reported by the DHS in 2012–2013 that stated that only 38% of women delivered in a health facility [10]. Also Morris and colleagues reported in a recent study that among 282 births delivered by 210 participants, only 39% were delivered in a hospital [25]. The most likely explanation for this difference was the fact that most of our study participants were recruited at the hospital or dispensary where they received services and might be more likely to have sought institutional delivery. Another important finding was that among the 79 women who reported an unplanned home birth the main reasons were precipitous labor and transportation issues (64 of 79 women). In the “Three Delays Model”, these correspond mainly to the second delay (delay due to distance and availability or cost of transport) but might also relate to poor understanding of when to seek medical help (first delay) [26]. Therefore, strengthening access to health care either by provision of health centers in rural and remote areas, introduction of maternity waiting homes and/or motorcycle ambulances could improve access.

Some limitations should be considered when interpreting the findings of our study. One is that our sample size was relatively small. Furthermore, causal relationships could not be established due to the cross-sectional study design. Moreover, most data were self-reported and not validated using objective measures. Interviewer and social desirability biases may also be possible, but are less likely in childbirth-related events compared with other more sensitive issues. Furthermore, we attempted to minimize the interviewer bias by employing local community interpreters speaking Sakalava. A recall bias, especially considering pregnancy intents but also the experience of the most recent birth might have also influenced the study results. However, our study included only women less than 13 months post-delivery. Therefore, we consider this effect relatively small. Furthermore, as the study has been conducted in between April to October 2015, it cannot be excluded that women have shared information of their education received about obstetric danger signs by the PANDA mHealth with other pregnant women. A strength of our study aside from the closeness to the birth was that danger signs were recorded unprompted by the interviewer.

## Conclusion

In this study a significant proportion of mothers in Ambanja, Madagascar, showed low knowledge of danger signs during delivery, the postpartum and in the newborn. Women who do not have the knowledge about obstetric danger signs may be more likely to delay seeking obstetric health care and are therefore at greater risk of maternal health complications. The receipt of information was found to be independent predictors of knowledge of danger signs during delivery and in the newborn. The participation in the mHealth intervention did not improve the knowledge of danger signs significantly, but showed a promising tendency.

The results of our study are among the first reported for Madagascar, but are consistent with other studies conducted in African countries that suggest that ANC has the potential to increase the awareness of obstetric danger signs in pregnant women. Therefore, health authorities and partner organizations working in the field of reproductive health should strengthen existing strategies to educate women during ANC visits, but also through public health campaigns. mHealth experts should consider improving the educational section of the the PANDA mHealth intervention and to test it on a larger scale.

## Additional file

Additional file 1: RENY_QuestionnaireFR (DOCX 118 kb)

## Abbreviations

ANC: Antenatal care
CI: Confidence intervals
CMC: Centre Médico-Chirurgical
mHealth: Mobile health
OR: Odds ratio
PANDA: Pregnancy-And-Newborn Diagnostic-Assessment
WHO: World Health Organization

## References

[1] Alkema L, Chou D, Hogan D, Zhang S, Moller AB, Gemmill A, Fat DM, Boerma T, Temmerman M, Mathers C, Say L, United Nations Maternal Mortality Estimation Inter-Agency Group collaborators and technical advisory group (2016). Global, regional, and national levels and trends in maternal mortality between 1990 and 2015, with scenario-based projections to 2030: a systematic analysis by the UN maternal mortality estimation inter-agency group. Lancet.

[2] WHO, UNICEF, UNFPA, World Bank Group, and United Nations population division maternal mortality estimation inter-agency group. Madagascar: Maternal mortality in 1990-2015. http://www.who.int/gho/maternal_health/countries/mdg.pdf. Accessed 23 Jan 2018.

[3] Say L (2014). Global causes of maternal death : a WHO systematic analysis. Lancet Glob Health.

[4] The World Health Report 2005. Make every mother and child count. World Health Organization 2005. http://www.who.int/whr/2005/whr2005_en.pdf. Accessed 09.01.2017.

[5] Benski AC, Stancanelli G, Scaringella S, Herinainasolo JL, Jinoro J, Vassilakos P, Petignat P, Schmidt NC (2017). Usability and feasibility of a mobile health system to provide comprehensive antenatal care in low-income countries: PANDA mHealth pilot study in Madagascar. J Telemed Telecare.

[6] Karkee R (2014). The role of obstetric knowledge in utilization of delivery service in Nepal. Health Educ Res.

[7] Hailu M, Gebremariam A, Alemseged F (2010). Knowledge About obstetric danger signs among pregnant women in Aleta Wondo district, Sidama zone, southern Ethiopia. Ethiop J Health Sci.

[8] Bogale D, Markos D. Knowledge of obstetric danger signs among child bearing age women in Goba district, Ethiopia: a cross-sectional study. BMC Pregnancy Childbirth. 2015;15(77) 10.1186/s12884-015-0508-1 PMC4381369 25886509

[9] WHO recommendations on antenatal care for a positive pregnancy experience. WHO 2016. http://apps.who.int/iris/bitstream/10665/250796/1/9789241549912-eng.pdf?ua=1, Accessed 19 Jan 2017. 28079998

[10] Institut National de la Statistique (INSTAT) et Partenaires Techniques et Financiers (PTF). 2014. Enquête Nationale sur le Suivi des OMD (ENSOMD) 2012–2013 Antananarivo, Madagascar. Available at: http://madagascar.unfpa.org/sites/default/files/pub-pdf/OMD_Resume.pdf. Accessed 14 June 2016.

[11] Kabakyenga J, Ostergren P-O, Turyakira E, et al. Knowledge of obstetric danger signs and birth preparedness practices among women in rural Uganda. Reprod Health. 2011;8(33) 10.1186/1742-4755-8-33 PMC3231972 22087791

[12] Pembe A, Urassa D, Carlstedt A (2009). Rural Tanzanian women’s awareness of danger signs of obstetric complications. BMC Pregnancy Childbirth.

[13] Hailu D, Berhe H (2014). Knowledge about obstetric danger signs and associated factors among mothers in Tsegedie district, Tigray region, Ethiopia 2013: community based cross-sectional study. PLoS One.

[14] Mutiso SM, Qureshi Z, Kinuthia J (2008). Birth Preparedness among antenatal clients. East Afr Med J.

[15] Nigatu SG, Worku AG, Dadi AF. Level of mother's knowledge about neonatal danger signs and associated factors in north west of Ethiopia: a community based study. BMC Res Notes. 2015;8(309) 10.1186/s13104-015-1278-6 PMC4506763 26188481

[16] Ekwochi U, Ndu IK, Osuorah CD, Amadi OF, Okeke IB, Obuoha E, Onah KS, Nwokoye I, Odetunde OI, Obumneme-Anyim NI. Knowledge of danger signs in newborns and health seeking practices of mothers and caregivers in Enugu state, south-East Nigeria. Ital J Pediatr. 2015;41(18) 10.1186/s13052-015-0127-5 PMC4372313 25888409

[17] Madagascar. Countdown to 2015. Maternal, Newborn and Child Survival. http://www.countdown2015mnch.org/documents/2013Report/Madagascar_Accountability_profile_2013.pdf. Accessed 23 Jan 2018.

[18] Gagnon AJ, Sandall J. Individual or group antenatal education for childbirth or parenthood, or both. Cochrane Database Syst Rev 2007. 2011;(3). Art. No.: CD002869. 10.1002/14651858.CD002869.pub2. 10.1002/14651858.CD002869.pub2 PMC6999801 17636711

[19] Amoakoh-Coleman M, Borgstein AB, Sondaal SF, Grobbee DE, Miltenburg AS, Verwijs M, Ansah EK, Browne JL, Klipstein-Grobusch K. Effectiveness of mHealth interventions targeting health care workers to improve pregnancy outcomes in low- and middle-income countries: a systematic review. J Med Internet Res. 2016;18(8):e226. 10.2196/jmir.5533 PMC5010646 27543152

[20] Sedgh G, Singh S, Hussain R (2014). Intended and unintended pregnancies worldwide in 2012 and recent trends. Stud Fam Plan.

[21] Institut National de la Statistique (INSTAT) et ICF Macro (2010). Enquête Démographique et de Santé de Madagascar 2008-2009.

[22] H S, Kabagenyi A, Anguzu R, Muhumuza C, Hassen K, Sudhakar M (2016). Family planning counseling during antenatal care and postpartum contraceptive uptake in Africa: a systematic review protocol. JBI Database System Rev Implement Rep.

[23] Perumal N, Cole DC, Ouédraogo HZ, Sindi K, Loechl C, Low J (2013). Health and nutrition knowledge, attitudes and practices of pregnant women attending and not-attending ANC clinics in western Kenya: a cross-sectional analysis. BMC Pregnancy Childbirth.

[24] World Health Organization and UNICEF 2012. Building a Future for women and children. The 2012 Report. https://www.unicef.org/eapro/Countdown_to_2015.pdf. Accessed 19 Jan 2017.

[25] Morris JL (2014). Maternal health practices, beliefs and traditions in Southeast Madagascar. Afr J Reprod Health.

[26] Thaddeus S, Maine D. Too far to walk: maternal mortality in context. Soc Sci Med. 38(8):1091, 1110. 10.1016/0277-9536(94)90226-7 8042057

