# Supplementary material for: Cross-sectional survey of knowledge of obstetric danger signs among women in rural Madagascar
Source: BMC Pregnancy Childbirth. 2018 Feb 5;18:46. doi: 10.1186/s12884-018-1664-x (PMC5800042; doi:10.1186/s12884-018-1664-x)
Supplement: Additional file 1: — RENY_QuestionnaireFR (DOCX 118 kb) [file 12884_2018_1664_MOESM1_ESM.docx]

**Questionnaire RENY VOARO**

.

| CODE participant : __/__/__/ |  |  |
| --- | --- | --- |
| Date : __ __/__ __/__ __ __ __ | Questionnaire sur les soins à l’accouchement | Page 1 de 11 |

Questionnaire sur les soins de santé à l’accouchement

Étude de cohorte menée dans la région d’Ambanja

1. Numéro de l’interviewer /___/___/
2. Initiales de l’interviewer /___/___/
3. Lieu de l’entrevue ___________________________________
4. Heure de l’entrevue /___/___/ : /___/___/
5. Participante de l’étude PANDA ? ☐ oui

☐ non

| **I : Facteurs sociodémographiques** | | | | | | | | | | | |  |  |
| --- | --- | --- | --- | --- | --- | --- | --- | --- | --- | --- | --- | --- | --- |
| Q1 | Quel âge avez-vous ? | _____________ans | | |  | | |  | | | |  |  |
| Q2 | À quand remonte votre dernier accouchement ?  *Insérer la date exacte si possible. Sinon, mois et année.* | ___/___/2015  ___/___/2014  Pendant ou avant 2013 **(EXCLUSION)** | | | 1  2  0  0 | | |  | | | |  |  |
| Q3 | Depuis combien de temps habitez-vous la région d’Ambanja? | Indiquer les années___________  *Si la patiente est née à Ambanja, passé à Question 5* | | | | | | | | | |  |  |
| Q4 | Où êtes-vous née? | Région ou ville de Madagascar  Autre pays ___________________ | | | | | | | | | |  |  |
| Q5 | Quel est votre niveau d’éducation le plus élevé ? | Aucun  École primaire  École secondaire 1  École secondaire 2  Études tertiaires  Je ne sais pas | | | 0  1  2  3  4  9 | | |  | | | |  |  |
| Q6 | Quelle est la plus haute classe atteinte dans ce niveau ? | Classe ________  Je ne sais pas | | | | | | | | | |  |  |
| Q7 | Quelle est votre profession ? | Femme au foyer  Fermière  Commerçante  Sans emploi  Autres,  précisez _______________ | | | 1  2  3  4  10 | | | | |  | |  |  |
| Q8 | Comment êtes-vous rémunérée pour votre travail ? | Argent (uniquement)  Argent et éléments naturels  Eléments naturels uniquement  Pas de rémunération | | | 1  2  3  4 | | | | |  | |  |  |
| Q9 | Quelle est votre situation familiale ? | Mariée  Célibataire  En cohabitation  Vit ensemble avec un partenaire  Séparée/divorcée  Veuve | | | 1  2  3  4  5  6 | | | | | →Q13  →Q13  →Q13  →Q13 | |  |  |
| Q10 | Quelle est la profession de votre mari ? | Fermier  Journalier  Commerçant  Sans emploi  Autre,  précisez ______________ | | | 1  2  3  4  10 | | | | |  | |  |  |
| Q11 | Comment est rémunéré votre mari/partenaire pour son travail ? | Argent (uniquement)  Argent et éléments naturels  Eléments naturels uniquement  Pas de rémunération | | | 1  2  3  4 | | | | |  | |  |  |
| Q12 | Quel est le niveau d’éducation de votre mari / partenaire? | Aucun  École primaire  École secondaire 1  École secondaire 2  Études tertiaires  Je ne sais pas | | | 0  1  2  3  4  9 | | | | |  | |  |  |
| Q13 | Quelle est votre religion ? | Aucune  Catholicisme  Protestantisme  Religion traditionnelle/animiste  Islam  Autre,  précisez _________________ | | | 0  1  2  3  4  10 | | | | |  | |  |  |
| Q14 | Combien de membres de votre famille habitent avec vous dans votre résidence ? | 1-3  4-5  6 ou plus | | | 1  2  3 | | | | |  | |  |  |
| Q15 | Où obtenez l’eau que vous buvez ? | Dans la maison  Dans la cour/la parcelle d’habitation  En dehors de l’habitation | | | 1  2  3  32  3 | | | | |  |  | |  |
| Q16 | Votre foyer a-t-il des toilettes privées ? Ou partagez-vous avec d’autres foyers ? | Toilettes privées  Toilettes partagées | | | 1  2 | | | | |  |  | |  |
| Q17 | Combien de chambres à coucher avez-vous ? | Une  Deux  Trois ou plus | | | 1  2  3 | | | | |  |  | |  |
| Q18 | Quel est le revenu mensuel de votre famille ? (en : Ariary malagache) | _____________________  Je ne sais pas | | | | | | | | | |  |  |
| Q19 | Avez-vous à la maison :  -électricité  -radio  -télévision  -téléphone fixe  -téléphone mobile  -réfrigérateur | OUI NON  électricité  radio  télévision  téléphone fixe  téléphone mobile  réfrigérateur | | | | | | | | | |  |  |
| Q20 | Qui prend la décision finale par rapport… | **….à votre santé :**  femme même  partenaire  les deux ensembles  quelqu’un autre, précisez  ______________________  **--- à des décisions quotidiennes à la maison :**  femme même  partenaire  les deux ensembles  quelqu’un autre, précisez  ______________________ | | | | | | | | | |  |  |
| **II Accès à un établissement de santé** | | | | | | | | | | | |  |  |
| Q21 | Y a-t-il un établissement de santé (ES) dans votre village ? | Non  Oui  Je ne sais pas | | | 0  1  9 | | | →Q24 | | | |  |  |
| Q22 | Quel type d’établissement de santé y a-t-il dans votre village ? | Hôpital  Dispensaire  Aucun | | | 1  2  0 | | |  | | | |  |  |
| Q23 | À quelle distance de marche se trouve l’établissement de santé le plus proche de votre résidence ? | <30 minutes  30 minutes — 1 heure  1-2 heures  >2 heures | | | 1  2  3  4 | | |  | | | |  |  |
| Q24 | Quel type de transport utilisez-vous pour atteindre la structure de santé durant le jour ou pendant la nuit ? (plusieurs réponses possibles) | JOUR NUIT  Bus  Vélo  Voiture  Pied  Cheval  Autre  *S’il vous plait à cocher tous les réponses qui sont présente.*  *Par ex. à pied possible pendant jour et nuit :*  JOUR NUIT  Pied  x  x  *Mais Bus que disponible pendant la journée :*  Bus  x | | | | | | | | | |  |  |
| **III Histoire obstétricale** | | | | | | | | | | | |  |  |
| Q25 | Pour la dernière grossesse, après combien de semaines ou mois de grossesse avez-vous accouché ? | Veuillez préciser  ______ semaines de grossesse  ______ mois de grossesse  ☐ Ne sais pas  Type de grossesse :  Grossesse simple  Grossesse multiple | | | 1  2 | | | | |  | |  |  |
| Q26 | Combien de fois êtes-vous tombée enceinte ? | 1-4  5-7  8-11  >11 | | | 1  2  3  4 | | | | |  | |  |  |
| Q27 | Combien de fois êtes-vous tombée enceinte dans les 3 dernières années ? | 1  2  3  Plus de 3 fois | | | 1  2  3  4 | | | | |  | |  |  |
| Q28 | Au moment où vous êtes tombée enceinte, vouliez-vous tomber  enceinte à ce moment-là, vouliez-vous attendre plus tard, ou  vouliez-vous ne pas/ne plus avoir d’enfants ? | À ce moment-là  Plus tard  Ne pas/plus avoir d’enfants | | | 1  2  3 | | | | |  | |  |  |
| Q29 | Combien d’enfants vivantes avez-vous ? | Indiquez le nombre  _____ Filles  _____ Garçons | | |  | | | | |  | |  |  |
| Q30 | Combien d’enfants de moins de  5 ans avez-vous ? | 1  2  3  4 ou plus de moins de 5 ans | | | 1  2  3  4 | | | | |  | |  |  |
| Q31 | Avez-vous déjà eu une grossesse qui s'est terminée par une  fausse-couche, un avortement ou un mort-né ? | Oui  Non | | 1  0 | | | | | *→Q33* | | |  |  |
| Q32 | *Si la réponse est OUI, ajouter* | - Nombre de FC : __ __   -combien de FC >1^er^ trimestre   - Nombre d’avortement :   -combien d’avortement >1^er^ trimestre :   - Nombre de mort-né ? - -spécifier pour chaque mort né à combien de mois : - ____________________ - ___________________ - ___________________ - ___________________ | | | | | | | | | |  |  |
| **IV Soins prénataux** | | | | | | | | | | | |  |  |
| Q33 | Durant votre dernière grossesse, avez-vous effectué des contrôles ? | Non  Oui | 0  1 | | | | *→Q42* | | | | |  |  |
| Q34 | Si oui, qui l’a fait ? | Docteur  Sage-femme/Infirmier (ère)  Intervenant en santé dans votre communauté  Accoucheuse traditionnelle  Ombiasas  Autres,  précisez ___________________ | 1  2  3  4  5  10 | | | |  | | | | |  |  |
| Q35 | Où avez-vous fait ces contrôles ? | A la maison  Autre maison  Hôpital  Dispensaire  Autres, précisez __________________ | 1  2  3  4  10 | | | |  | | | | |  |  |
| Q36 | Durant quel mois de votre grossesse avez-vous eu le premier contrôle ? | Durant le_______^er/eme^ mois de grossesse  Je ne sais pas | 98 | | | |  | | | | |  |  |
| Q37 | Combien de fois avez-vous eu un contrôle programmé pendant la grossesse ? *(non-urgent)* | ________ fois  ☐ Je ne sais pas | 98 | | | |  | | | | |  |  |
| Q38 | A été effectué durant un contrôle : | OUI NON  Poids corporel  Prise de la tension artérielle  Test urinaire  Test sanguin | | | | |  | | | | |  |  |
| Q39 | Vous a-t-on conseillé un endroit où accoucher ? | Oui  Non | 1  0 | | | | *→Q43* | | | | |  |  |
| Q40 | Si oui : quel endroit vous a-t-on conseillé ? | Hôpital  Dispensaire  Autres, précisez _________ | 1  2  10 | | | |  | | | | |  |  |
| Q41 | Quels arguments avez-vous reçus en faveur d’un accouchement dans l’établissement de santé ?  (réponse libre)  *Essayer de voir si la patiente vous donner librement une explication, seulement à cocher si elle dit qu’elle ne sait pas ou qu’elle n’a pas reçue un argument.* | _______________________________________________  ☐ Je ne sais pas  ☐Pas d’argument donné. | | | | | | | | | |  |  |
| Q42 | Si vous n’avez pas eu de contrôle pendant la grossesse, quelle en était la raison ?  *Possibilité de cocher plusieurs propositions, mais limité à 3 le plus important. Réponse libre.* | Je n’avais pas de problèmes de santé  J’avais trop de travail à faire  L’établissement de santé était trop loin  Mon mari a refusé  J’avais peur des coûts à payer  Je n’aimais pas l’établissement de santé  J’avais honte  Je ne savais pas où aller  Autre, précisez_________________ |  | | | |  | | | | |  |  |
| ***Ci-dessous, nous vous demanderons plusieurs questions à propos des femmes enceintes et de l’accouchement en général mais aussi à propos de vos propres expériences. Nous aimerions vous rappeler que nous ne faisons pas partie des professionnels de la santé à Madagascar. Les informations récoltées resteront confidentielles et n’influenceront pas votre prise en charge médicale…*** | | | | | | | | | | | |  |  |
| **V.1 Attitudes et perceptions** | | | | | | | | | | | |  |  |
| Q43 | Croyez-vous qu’il y ait une différence entre accoucher à la maison et accoucher dans un établissement de santé ? | Oui  Non  Je ne sais pas | | | 1  0  9 | | |  | | | |  |  |
| Q44 | Pourquoi pensez-vous qu’une femme accouche à la maison ?  *(Possibilité de cocher plusieurs propositions. Cocher en fonction des réponses de la participante SANS lire la liste. En cas de >3 réponses, demander les 3 causes le plus important pour elle.)* | La famille est présente  Il y a plus d’intimité  Par décision de la famille ou du mari  Pour le respect des coutumes  La qualité des soins à l’établissement de santé est mauvaise  Le personnel de santé est peu qualifié  L’accouchement est facile  A cause d’un problème de transport  L’établissement de santé est trop loin  Par peur des coûts à payer  Autre, précisez _______________  Pas de réponse de la femme à cette question. | | |  | | |  | | | |  |  |
| Q45 | Pourquoi pensez-vous qu’une femme accouche dans un établissement de santé (ES)?  *(Possibilité de cocher plusieurs propositions. Cocher en fonction des réponses de la participante SANS lire la liste.)* | Les ES sont plus propres  Les ES peuvent sauver la vie des mères  Les ES peuvent sauver la vie des bébés  L’accouchement dure moins longtemps dans les ES  Autre, précisez_____________  Pas de réponse de la femme à cette question. | | |  | | |  | | | |  |  |
| Q46 | *Maintenant on aimerait parler de vous-même :*  Où avez-vous accouché pour votre dernier enfant ? | À la maison  Dans un hôpital  Dans un dispensaire  Autre,  précisez__________________ | | | 1  2  3  10 | | | →Q51  →Q51  →Q51 | | | |  |  |
| Q47 | Est-ce que vous aviez prévu d’accoucher à la maison ? | Oui  Non | | | 1  0 | | | →Q49  →Q48 | | | |  |  |
| Q48 | Si vous avez accouché à la maison et ce n’était pas prévu, pourquoi ? | Accouchement imminent, pas de temps pour aller dans un ES  A cause d’un problème de transport  Par décision de la famille ou du mari  Autre, précisez_________________ | | | 1  2  3  10 | | |  | | | |  |  |
| Q49 | Pourquoi avez vous choisi d’accoucher à domicile pour votre dernière grossesse ? | Accouchement précédent facile  Il y a plus d’intimité  Par décision de la famille ou du mari  Pour le respect des coutumes  A cause d’un problème de transport  L’établissement de santé est trop loin  Par peur des coûts à élevés  Autre, précisez _______________ | | | 1  2  3  4  5  6  7  10 | | |  | | | |  |  |
| Q50 | Qui vous a aidé lors de votre dernier accouchement ?  ***Plusieurs réponses possibles.*** | Mère  Belle-mère  Accoucheuse traditionnelle  Voisin(e)  Docteur  Sage-femme  Je ne sais pas/je ne me souviens pas  Autre, précisez _________________ | | | 1  2  3  4  5  6  9  10 | | |  | | | |  |  |
| Q51 | Est-ce qu’il y avait d’autres personnes présentes lors du dernier accouchement ? | Personne  Mari  Mère  Belle-mère  Quelqu’un d’autre, spécifiez  _______________ | | | 1  2  3  4  10  1  2  3  4 | | | | |  |  | |  |
| Q52 | Quelle coutume avez-vous pu faire à la maison que vous n’auriez pas pu faire dans un ES ?  *Réponse libre.* | _____________________________________________  _____________________________________________  _____________________________________________ | | | | | | | | | |  |  |
| Q53 | Si vous avez donné naissance à votre dernier enfant dans un établissement de santé, pourquoi ?  ***(Possibilité de cocher plusieurs propositions. Cocher en fonction des réponses de la participante sans lire la liste)*** | J’étais malade  Les services sont bons  Les services sont gratuits  L’établissement de santé est proche  Les établissements de santé aident à sauver la vie des mères  J’ai eu une mauvaise expérience lors d’une grossesse précédente  L’éducation à la santé que j’ai reçue m’a encouragée à accoucher dans un établissement de santé  Autre, précisez _________________ | | |  | | | | |  | |  |  |
| Q54 | Qui a décidé où vous alliez accoucher ? | Moi-même  Mari  Mari et moi-même  Mère/Belle-mère  Professionnel de la santé  Autre, précisez_____________ | | | 1  2  3  4  5  10 | | | | |  | |  |  |
| Q55 | Combien de temps a duré votre dernier accouchement ?  ***(A demandé la participantes combien des heures à duré son accouchement et classifier vous-même.)*** | <2h  2-12 h  >12 h  Je ne sais pas/je ne me souviens pas | | | | 1  2  3  9  1 | | | |  |  | | |
| Q56 | Si vous avez donné naissance à votre dernier enfant dans un établissement de santé, qui a vous aidé lors de votre dernier accouchement ?  ***Plusieurs réponses possibles*** | Docteur  Sage-femme  Je ne sais pas/je ne me souviens pas  Autre, précisez _________________ | | | 1  2  9  10 | | | | |  | |  |  |
| **V.2 Complications grossesse et accouchement : connaissances personnelles**  **A mettre par l’interviewer s’il y avait un module éducatif au début de l’entretien :** oui/non | | | | | | | | | | | |  |  |
| Q57 | A votre avis, peut-il y avoir des problèmes durant la grossesse ou l’accouchement qui mettent en danger la vie de la femme? | Oui  Non  Ne sais pas | | | | 1  0  9 | | | |  | |  |  |
| Q58 | Avez-vous déjà reçu des informations à propos des problèmes pendant la grossesse ou l’accouchement? | Oui  Non | | | | 1  0  1 | | | | →Q60 | |  |  |
| Q59 | Si oui, qui vous les a données ? | Docteur  Sage-femme/Infirmier (ère)  Intervenant en santé dans votre communauté  Amis/Voisins/Famille  Radio  Autres____________ | | | | 1  2  3  4  5  10  9 | | | |  | |  |  |
| Q60 | D’après vous, quels sont les signes de danger les plus communs pendant la grossesse?  ***(Possibilité de cocher plusieurs propositions. Cocher en fonction des réponses de la participante SANS lire la liste)*** | Saignements vaginaux  Mains et corps gonflés  Perte de connaissance et convulsions  Vision trouble  Maux de tête violents ou vertiges  Fièvre  Douleur abdominale aigüe  Pas de mouvement du bébé dans le ventre  Autre, précisez ____________  Pas de réponse de la femme à cette question. | | | | | | | | | |  |  |
| Q61 | Quels sont les signes de danger les plus communs durant le travail et l’accouchement ?  ***(Possibilité de cocher plusieurs propositions. Cocher en fonction des réponses de la participante SANS lire la liste)*** | Saignement vaginal sévère  Travail prolongé de plus de 12h  Placenta non expulsé 30 min après l'accouchement  Perte de connaissance et convulsions  Autres, précisez____________ | | | | 1  2  3  4  10  2  3 | | | |  | |  |  |
| Q62 | Quels sont les signes de danger les plus communs durant le premier mois après l’accouchement chez la mère ?  ***(Possibilité de cocher plusieurs propositions. Cocher en fonction des réponses de la participante SANS lire la liste)*** | Saignement vaginal sévère  Fièvre  Pertes vaginales malodorantes  Mains et corps gonflés  Perte de connaissance et convulsions  Autre,  précisez____________ | | | | 1  2  3  4  5  10  2  3  4  5 | | | |  | |  |  |
| Q63 | Quels sont les signes de danger pour le nouveau-né ?  ***(Possibilité de cocher plusieurs propositions. Cocher en fonction des réponses de la participante SANS lire la liste)*** | Ne tète pas, a de la peine à manger ou vomit  A de la peine à respirer  Bébé de couleur bleu  Est froid ou chaud ou avec une fièvre sévère  Eruption cutanées ou boutons  Tout petit bébé  Autre, précisez  _____________________  ____________________ | | | | 1  2  3  4  5  6  10  1  3  4  5  6  7 | | | |  | |  |  |
| Q64 | Est-ce que vous pouvez nous expliquer qu’est-ce que vous avez fait après la naissance pour rester en bonne santé ?  *Réponse libre.* | ____________________________________  ____________________________________  Pas de réponse de la femme à cette question. | | | | | | | | | |  |  |
| **VI. Complications grossesse et accouchement : expériences personnelles** | | | | | | | | | | | |  |  |
| Q65 | Avez-vous eu un contrôle dans les 2 premiers mois après votre accouchement ? | Oui  Non | | | 1  0 | | |  | | | |  |  |
| Q66 | Vous-même avez-vous eu des problèmes durant un accouchement ? | Oui  Non | | | 1  0 | | | →Q68 | | | |  |  |
| Q67 | **Si oui**, quelle sorte de problème ?  ***(Possibilité de cocher plusieurs propositions. Cocher en fonction des réponses de la participante SANS lire la liste)*** | Saignement vaginal sévère  Travail prolongé de plus de 12h  Placenta non expulsé 30 min après l'accouchement  Perte de connaissance et convulsions  Fœtus d’apparence anormale  Décès du fœtus dans l’utérus  Autre,  précisez_____________ | | | 1  2  3  4  5  6  10 | | |  | | | |  |  |
| Q68 | Est-ce qu’un de vos bébés a déjà eu des problèmes dans les premières heures suivant un accouchement ? | Oui  Non | | | 1  0 | | | →Q71 | | | |  |  |
| Q69 | Si oui, quelle sorte de problèmes ?  ***(Possibilité de cocher plusieurs propositions. Cocher en fonction des réponses de la participante SANS lire la liste)*** | Le bébé ne respirait pas  Le bébé ne pleurait pas  Le bébé ne voulait pas manger  Le bébé était très bleu  Le bébé était froid  Le bébé est mort peu après l’accouchement  Autre,  précisez_____________ | | | 1  2  3  4  5  6  9 | | |  | | | |  |  |
| Q70 | Si oui, étiez-vous à la maison ou dans un établissement de santé ? | Maison  Etablissement de santé  Autre,  précisez_____________ | | | 1  2  10 | | |  | | | |  |  |
| Q71 | Avez-vous eu des problèmes dans les 2 premiers mois après l’accouchement ?  ***(Possibilité de cocher plusieurs propositions. Cocher en fonction des réponses de la participante SANS lire la liste)*** | Pas de problème  Saignement vaginal sévère  Travail prolongé de plus de 12h  Placenta non expulsé 30 min après l'accouchement  Perte de connaissance et convulsions  Décès du fœtus dans l’utérus  Autre,  précisez_____________ | | | 01  2  3  4  5  10 | | |  | | | |  |  |
| Q72 | Où préfèreriez-vous accoucher la prochaine fois ? | Maison  Etablissement de santé | | | 1  2 | | |  | | | |  |  |
| Q73 | Si vous tombez enceinte à nouveau, par qui désireriez-vous être aidée ? | Mère  Belle-mère  Accoucheuse traditionnelle  Voisin(e)  Docteur  Sage-femme  Je ne sais pas/je ne me souviens pas  Autre, précisez _____________ | | | 1  2  3  4  5  6  9  10 | | |  | | | |  |  |
| Q74 | Quant à votre mari, où préfère-t-il que vous accouchiez la prochaine fois ? | À la maison  Dans un établissement de santé  Autre, précisez _____________ | | | 1  2  10 | | |  | | | |  |  |

**MERCI POUR VOTRE PARTICIPATION**
